# A Halogen-Containing Stilbene Derivative from the Leaves of *Cajanus cajan* that Induces Osteogenic Differentiation of Human Mesenchymal Stem Cells

**DOI:** 10.3390/molecules200610839

**Published:** 2015-06-11

**Authors:** Jia-Zhong Cai, Rong Tang, Gui-Fu Ye, Sheng-Xiang Qiu, Nen-Ling Zhang, Ying-Jie Hu, Xiao-Ling Shen

**Affiliations:** 1 Laboratory of Chinese Herbal Drug Discovery, Tropical Medicine Institute, Guangzhou University of Chinese Medicine, Guangzhou 510405, China; E-Mails: jiazhongcai@gzucm.edu.cn (J.-Z.C.); tangrong0319@163.com (R.T.); yeguifu0120@163.com (G.-F.Y.); 2 Program for Natural Product Chemical Biology, Key Laboratory of Plant Resources Conservation and Sustainable Utilization, South China Botanical Garden, Chinese Academy of Sciences, Guangzhou 510650, China; E-Mails: sxqiu@scbg.ac.cn (S.-X.Q.); zhangnenling@scbg.ac.cn (N.-L.Z.); 3 Pi-Wei Institute, Guangzhou University of Chinese Medicine, Guangzhou 510405, China

**Keywords:** *Cajanus cajan* (L.) Millsp., stilbene, halogen, cajanstilbene H, osteogenic diffrentiation, human mesenchymal stem cells

## Abstract

A new natural halogen-containing stilbene derivative was isolated from the leaves of *Cajanus cajan* (L.) Millsp. and identified as 3-*O*-(3-chloro-2-hydroxyl-propanyl)-longistylin A by comprehensive spectroscopic and chemical analysis, and named cajanstilbene H (**1**). It is the first halogen-containing stilbene derivative found from plants. In human mesenchymal stem cells (hMSC) from bone marrow, **1** did not promote cell proliferation, but distinctly enhanced osteogenic differentiation of hMSC in time- and dose-dependent manners. In six human cancer cell lines, 1 showed a moderate inhibitory effect on cell proliferation, with IC_50_ values of 21.42–25.85 μmol·L^−1^.

## 1. Introduction

*Cajanus cajan* (L.) Millsp., commonly known as pigeon pea, is a plant of Fabaceae grown in semiarid tropics in Asia and Africa. Extracts and components from pigeon pea leaves were reported to show a variety of bioactivities, including antimalaria [1], cytotoxicity [2,3], hypolipidemia [4], reducing bone loss and promoting bone-like tissue formation [5,6,7]. A series of phytochemical studies have demonstrated that flavonoids and stilbenoids are main chemical constituents of the leaves [8,9,10,11,12,13,14]. In our previous investigation, a hydrophobic fraction of pigeon pea leaves showed activity in increasing tibial bone density and improving bone metabolism and lipid metabolism in *ob*/*ob* mice with osteoporosis and hyperlipidemia symptom [15]. In this paper, we present the identification of a new compound from this extract and its bioactivities in oesteogenic differentiation of human mesenchymal stem cells (hMSC) from bone marrow and cytotoxicity against human cancer cell lines.

## 2. Results and Discussion

### 2.1. Structural Elucidation of Compound ***1***

**Figure 1:**
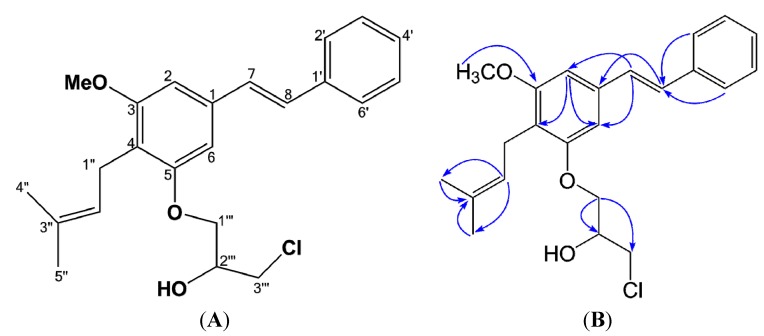
Structure (**A**) and Key HMBC correlations (**B**) of cajanstilbene H (**1**).

*Cajanstilbene H* (**1**) was obtained as colorless amorphous powder. The 50% alcoholic solution gave negative color reaction upon addition of FeCl_3_ reagent. UV (MeOH) λ_max_ (log *ε*) 211 (2.12), 236 (1.10), 317 (2.09) nm; IR (KBr) ν_max_ 3401, 3025, 2917, 1600, 1575, 1450, 1417, 1168, 1118, 956 cm^−1^; EIMS *m/z* (relative abundance; %): 388 (32.22), 386 (100.00), 373 (21.50), 371 (64.99), 357 (1.39), 355 (4.09), 333 (3.39), 331 (10.17), 320 (3.60), 318 (10.61), 295 (4.16), 293 (22.04), 279 (13.35), 277 (36.82). The chlorine-containing molecular feature of **1** was suggested upon the characteristic fragmentation pattern in EIMS *m/z* (relative abundance; %): 388 (32.22), 386 (100.00), 373 (21.50), 371 (64.99), 357 (1.39), 355 (4.09), 333 (3.39), 331 (10.17), 320 (3.60), 318 (10.61), wherein ion pairs with difference of 2 mass units ([m + 2]/[m]) were observed in a 1:3 abundance ratio. Molecular formula of **1** was determined as C_23_H_27_O_3_Cl (*m/z* 386.1634, calcd. for C_23_H_27_O_3_Cl, 386.1643) by HR-MS, which was proved by the abundance ratio from molecular ion (*m/z* 386.1634, 100.0%) and its [M + 2] fragment (*m/z* 388.1613, 34.8%), and the presence of chlorine assayde by ion chromatography (Supplemental Information, Pages S11). The NMR spectral data of **1** (Table 1) were similar to those of longistylin A, a major stilbene previously isolated from the same plant [1,10], suggesting the existence of a longistylin A moiety. A closer inspection of the NMR spectra revealed the subtle differences between longistylin A and **1**: the resonance signal of C-4 shifted downfield from δ 115.0 in longistylin A to δ 118.3 in **1**, while the resonance of C-6 shifted upfield from δ 107.1 in longistylin A to δ 102.9 in **1**, due to the shift-induced effects by C-5 substitution; consistently, a proton signal at δ 5.29 (1H, s) ascribing to 5-OH of longistylin A disappeared, but instead signals for a three-carbon aliphatic chain at δ_C_ 70.0 (CH), 68.9 (CH_2_) and 45.8 (CH_2_) respectively, were observed, indicating that **1** was a 5-*O*-substituted derivative of longistylin A. The ^13^C signals for the aliphatic chain correlated with proton signals at δ_H_ 4.21 (1H, m), 4.14 (2H, m) and 3.75 (2H, m) by ^13^C-^1^H COSY respectively, suggest the existence of a 3-chloro-1, 2-propanediol unit linked upon 5-OH group. Thus, the structure of **1** was deduced as 5-*O*-(3-chloro-2-hydroxylpropanyl)-longistylin A (Figure 1) and confirmed by the HMBC correlations (Table 1 and Figure 1). Compound **1** was the first halogen-containing stilbene derivative isolated from plant, then named as cajanstilbene H.

**Table 1:** ^1^H- and ^13^C-NMR data of **1** in CDCl_3_.

Position	Cajanstilbene H (1)		
	δ_C_^a^, type	δ_H_^b^ (*J* in Hz)	HMBC ^c^
1	136.4 s		7, 8
2	103.0 d	6.68 s	6, 7, 8
3	156.8 s		6
4	118.3 s		2, 6
5	158.2 s		2, OMe
6	102.9 d	6.71 s	2, 7, 8
7	128.8 d	7.04 s	2, 6, 2′, 3′, 5′, 6′
8	128.8 d	7.04 s	2, 6, 2′, 3′, 5′, 6′
1′	137.2 s		7, 8, 3′, 5′
2′, 6′	126.6 d	7.50 d (7.5)	7, 8, 2′, 4′, 6′
3′, 5′	128.7 d	7.35 t (7.5)	2′, 3′, 5′, 6′
4′	127.7 d	7.24 m	2′, 6′
1″	22.4 t	3.34 d (6.8)	
2″	123.2 d	5.11 t (6.8)	
3″	131.5 s		4′′, 5′′
4″	25.7 q	1.66 s	2′′
5″	17.9 q	1.79 s	2′′
5-OMe	55.8 q	3.86 s	
3-OH			
1′′′	68.9 t	4.14 m	3′′′
2′′′	70.0 t	4.21 m	1′′′, 3′′′
3′′′	45.8 t	3.75 m	1′′′
2′′′-OH		2.70 s	

^a^ Measured at 400 MHz. ^b^ Measured at 100 MHz. ^c^ HMBC corrections: H to C. Assignments were supported with HSQC and HMBC spectra.

Chemically, the structure of **1** could be formed by condensation of longstylin A and 3-monochloro-1, 2-propanediol (3-MCPD) with loss of one molecule of water under acidic condition. Compound **1** on earth is a natural product or an artificial product formed in extraction is an interesting question. 3-MCPD usually exists in form of fatty acid ester [16]; however, solvents used in extraction and isolation do not include oil, fatty acid ester, and glycerol or propanediol, which might be contaminated with 3-MCPD. Therefore, 3-MCPD should not be mixed in solvents. Moreover, because a hypothetic condensed product of 3-MCPD with longstylin C, a major stilbene in leaves of pigeon pea with higher quantity [17], less steric hinderance, and more favorable reactivity as compared to longstylin A, was not isolated, implying **1** should be a biosynthesized product of the plant and some stereospecific enzyme(s) may be involved in the biosynthesis.

### 2.2. Osteogenic Differentiation of hMSC

**Figure 2:**
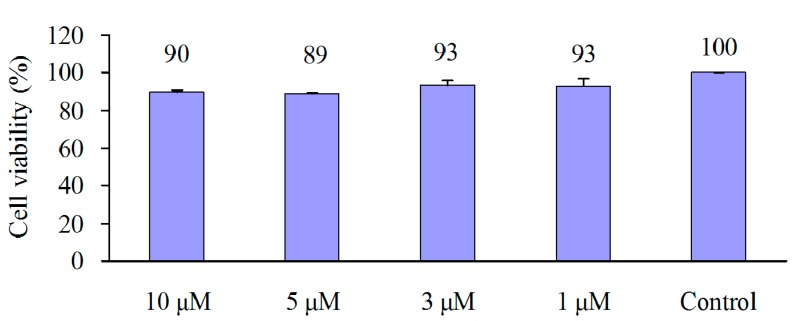
The 48 h proliferation assay for compound **1** in human mesenchymal stem cells (hMSC). Cell viability was measured using Cell Conuting Kit 8. Data were expressed as Mean ± SD (*n* = 3).

**Figure 3:**
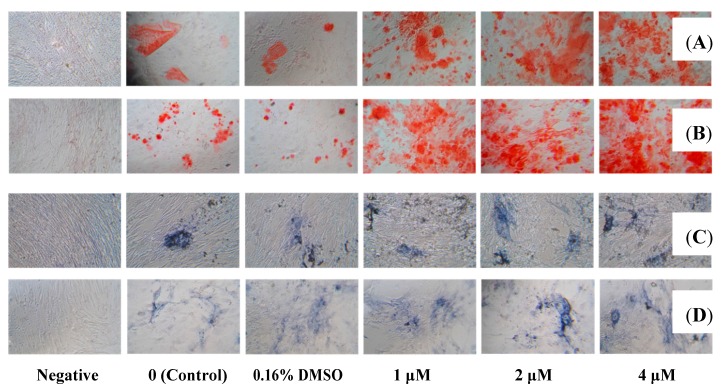
Detection of extracellular calcium deposits and membrane alkaline phosphatase (AP) activity in differentiated osteoblasts. Alizarin red S staining after inducing for osteogenic differentiation for 14 days (**A**) and 17 days (**B**) showed time and dose-dependent increases in calcium deposits (red color) in hMSC treated with compound **1**, indicating enhanced osteoblast formation. 5-Bromo-4-chloro-3-indolyl phosphate/Nitro blue tetrazolium (BCIP/NBT) staining (cell AP activity staining kit) ((**C**) 14 days; (**D**) 17 days) exhibited much more blue-violet color in hMSC treated with **1** than that in control or cells treated with 0.16% DMSO, inferring elevated cellular AP activity in osteoblasts.

Extracellular calcium deposits are the indication of successful differentiation of MSC into osteoblasts and *in vitro* bone-formation. Calcium deposits can be detected after being stained bright orange-red with Alizarin Red S [18]. Highly expressed membrane alkaline phosphatase (AP) is another indication of osteoblast [19], which can be easily detected using 5-Bromo-4-chloro-3-indolyl phosphate/Nitro blue tetrazolium (BCIP/NBT) to stains cells blue-violet when AP is present [20]. In this study, the effect of **1** on osteogenesis was investigated by employing hMSC, the precurcer cell of osteoblast. Results revealed that, incubation of **1** at concentrations of 10–1 μmol·L^−1^ together with hMSC in MSC growth medium for 48 h did not increase cell viability (Figure 2), but incubation of **1** (4, 2 or 1 μmol·L^−1^) together with hMSC in osteogenic differentiation medium achieved dramatic increases in extracellular calcium deposits (Figure 3A,B) and membrane AP (Figure 3C,D), inferring that **1** did not promote proliferation but distinctly promoted osteoblast differentiation in hMSC in dose- and time-dependent manners. Zheng *et al.* showed that a lipophilic extract from *Cajanus cajan* could reduce bone loss in ovariectomy-induced bone loss rats [5], and 2-carboxyl-longistilin A, a known stilbene, is able to promote osteoblast cells proliferation and mineralize bone-like tissue formation in human osteoblast-like TE85 cells [6]. Our data provided evidence for the new compound cajanstilbene H (**1**) in potential use in anti-osteoporosis.

### 2.3. Cytotoxicity of **1** in Six Human Cancer Cell Lines

Inhibitory effects of **1** on human tumor cell proliferation were investigated in lung cancer cell line (NCI-H460), prostate cancer cell line (PC-3), breast cancer cell line MCF-7, cervical cancer cell line (HeLa), colorectal cancer cell line (HCT-15) and P-glycoprotein overexpressing multidrug resistant oral epidermoid carcinoma cell line (KB-V1). Cells were treated with different concentrations of **1** or paclitaxel (positive control) for 48 h. Table 2 shows the measured concentrations of **1** to inhibit 50% of cell viability (IC_50_), moderate growth inhibitory effects were observed for **1** in all six tested cancer cell lines, with IC_50_ values of 21.42–25.85 μM.

**Table 2:** IC_50_ values (μM) of **1** in six human cancer cell lines at 48 h. Cancer cells were treated with Cajanstilbene H (**1**) or paclitaxel (positive control) for 48 h. Cell viability was measured by MTT assay. Data were expressed as Mean ± SD of three independent experiments.

	NCI-H460	PC-3	MCF-7	HeLa	HCT-15	KB-V1
**1**	21.47 ± 3.14	25.83 ± 3.64	21.42 ± 3.31	25.85 ± 2.58	24.81 ± 5.20	22.29 ± 6.39
Paclitaxel	0.036 ± 0.004	0.048 ± 0.013	0.032 ± 0.004	0.072 ± 0.018	0.021 ± 0.003	0.225 ± 0.059

## 3. Experimental Section

Melting points were measured on a Pyris diamond differential scanning calorimeter (Perkin Elmer, Waltham, UK). UV spectra were recorded on a TU-1901 UV spectrometer (Purkinje General, Beijing, China). IR spectra were collected on a Bruker Equinox 55 FTIR/FTNIR Spectrometer (Bruker, Billerica, MA, USA). ^1^H-NMR, ^13^C-NMR, ^13^C-^1^H COSY, ^1^H-^1^H COSY and heteronuclear multiple bond correlation (HMBC) spectra were recorded on a Bruker DRX-400 instrument (Bruker, Billerica, MA, USA) using TMS as internal standard. ESIMS spectra were run on a MDS Sciex API 2000 LC/MS/MS system (MDS Sciex, Ottawa, Canada). EIMS and high resolution (HR)-EIMS data were obtained on a DSQ and a MAT95XP mass spectrometer (Thermo Finnigan, Waltham, USA). Ion chromatography was carried out with a DX-600 IC system (Dionex, Sunnyvale, CA, USA). Silica gel (200–300 meshes, Qingdao Marine Chemical Ltd., Qingdao, China), ODS-AP (40–60 μm, DAISO Co. Ltd., Hiroshima, Japan), and Sephadex LH-20 (GE Healthcare, Buckinghamshire, UK) were used for column chromatography.

Human mesenchymal stem cells isolated from the bone marrow of a 62-year-old male cacausian donor were purchased from PromoCell Biotech Co., Heidelberg, Germany. MSC growth medium and MSC osteogenic differentiation medium were also PromoCell products. Alizarin red S staining kit and Cell AP activity assay kit were from Nanjing Jiancheng Bioengineering Institute (Nanjing, China), Cell Counting Kit 8 (CCK-8) was the product of Dojindo Molecular Technologies, Inc. (Kumamoto, Japan), RPMI 1640, fetal bovine serum (FBS) were Gibco products (Rockville, MD, USA).

### 3.1. Plant Material and Extraction

Leaves of *Cajanus cajan* were collected from Wenshan County, Yunnan Province, P.R. China, in August, 2010 and authenticated by Professor Fu-Wu Xing (South China Botanical Garden, Chinese Academy of Sciences). A voucher specimen (No.SD20100802) was deposited at the Laboratory of Chinese Herbal Drug Discovery, Tropical Medicine Institute, Guangzhou University of Chinese Medicine. Air-dried leaves of *C. cajan* (100 kg) were extracted with 95% EtOH under reflux. The concentrated ethanolic extract was scattered with 60 °C hot water (200 L), and placed at room temperature (26 °C) for 10 h. After centrifugation, the precipitate was mixed with water (10 L) and extracted with CHCl_3_ sufficiently. The CHCl_3_-soluble extract (410 g) was subjected to silica gel column and eluted with petrol ether (PE), PE-CHCl_3_ (50:50 to 10:90, *v*/*v*), and PE-acetone (99:1 to 80:20) to afford five fractions (A−E). Fraction A (25 g), an elution of PE-CHCl_3_ (50:50), was separated on a Silica gel column eluting with PE-acetone (98:2) to provide cajanolactone A [12] (650 mg) and naringenin-4′,7-dimethyl ether [11] (10 g). Longistylin A [1,10] (680 mg) and longistylin C [1,10] (1.8 g) were obtained by recrystallization of fraction B and C, elutions of PE-CHCl_3_ (30:70) and (10:90). Fraction D, an elution of PE-acetone (90:10), was further purified on a silica gel column with elution of PE-EtOAc (95:5 to 85:15) to give five subfractions (D1–D5). Subfraction D2, an elution of PE-EtOAc (90:10), was re-chromatographed successively on silica gel and sephadex LH-20 columns, eluting with PE-EtOAc (90:10 and 93:7) and methanol, to afford pinostrobin [10] (1.1 g) and **1** (605 mg).

### 3.2. Proliferation and Osteoblast Differentiation in hMSC

To investigate the effect of **1** on h-MSC proliferation, 3000 cells in 100 μL MSC growth medium were seeded in 96-well plates and incubated for 24 h. Different concentrations of **1** were added and incubated for 48 h. CCK-8 (10 μL per well) was then added to cells to react for 2 h. The amount of water soluble pigment which was positively correlated with the number of living cell was evaluated by the optical density (OD) at 450 nm. Cell viability (CV) under different treatment was calculated by equation CV = OD_Treatment_/OD_Control_, in which the control is the well without drug treatment.

To investigate the effect of **1** on osteoblast differentiation, about 3000 hMSC in MSC growth medium were seeded in 96-well plates to grow to 100% confluency. Osteoblast differentiation was induced by incubating cells in MSC osteogenic differentiation medium for 14 or 17 days, with or without the existence of 4, 2 or 1 μmol·L^−1^ of **1**, or 0.16% DMSO. Medium was changed every 3 days. Cells treated with MSC growth medium were set as negative control. Osteoblast detection was performed by staining the extracellular calcium deposits bright orange-red with Alizarin Red S, or by staining membrane AP blue-violet with BCIP/NBT, by following the procedure of Alizarin Red S staining kit or cell AP activity staining kit, respectively.

### 3.3. Proliferation in Tumor Cell Lines

NCI-H460, PC-3 and HeLa cell lines were maintained in RPMI 1640 containing 10% FBS, MCF-7 and KB-V1 cell lines were maintained in DMEM containing 10% FBS, while HCT-15 cell line was maintained in RPMI 1640 containing 10% FBS, 2 mmol·L^−1^ l-glutamine, 1.5 g·L^−1^ sodium bicarbonate, 4.5 g·L^−1^ glucose, 10 mmol·L^−1^ HEPES and 1.0 mmol·L^−1^ sodium pyruvate. All cells were incubated at 37 °C, 5% CO_2_, saturated humidity. To maintain drug resistance of KB-V1, 200 μg·L^−1^ of vinblastine was added to the culture medium, and cells must be grown in paclitaxel free medium for 7 days before any experiment.

To investigate the inhibitory effect of **1** on tumor cell proliferation, cells in 100 μL growth medium were seeded in 96-well plates at a density of 5000 cells per well and incubated for 24 h to let cells to adhere. Cells were replaced the medium with fresh medium containing different concentrations of **1** or paclitaxel and incubated for 48 h. Cell viability under different treatment was measured by MTT assay and expressed as expressed as percentage of the control [21].

## 4. Conclusions

In our study, cajanstilbene H, a new halogen-containing stilbene derivative was identified from the leaves of pigeon pea. To our knowledge, it is the first naturally existing ether derivative of 3-monochloro-1,2-propanediol (3-MCPD). Importantly, cajanstilbene H strongly promoted osteoblast differentiation in hMSC, exhibiting a potential to alleviate osteoporosis.

## Supplementary Materials

The UV, IR, EIMS and HR-EIMS, 1D and 2D NMR spectra, ion chromatography of **1** are available, in the online version, at http://www.mdpi.com/1420-3049/20/06/10839/s1.
