# Supplementary material for: A Halogen-Containing Stilbene Derivative from the Leaves of Cajanus cajan that Induces Osteogenic Differentiation of Human Mesenchymal Stem Cells
Source: Molecules. 2015 Jun 11;20(6):10839–47. doi: 10.3390/molecules200610839 (PMC6272782; doi:10.3390/molecules200610839)
Supplement: Supplementary file 1 [file molecules-20-10839-s001.pdf]

Supplementary Materials

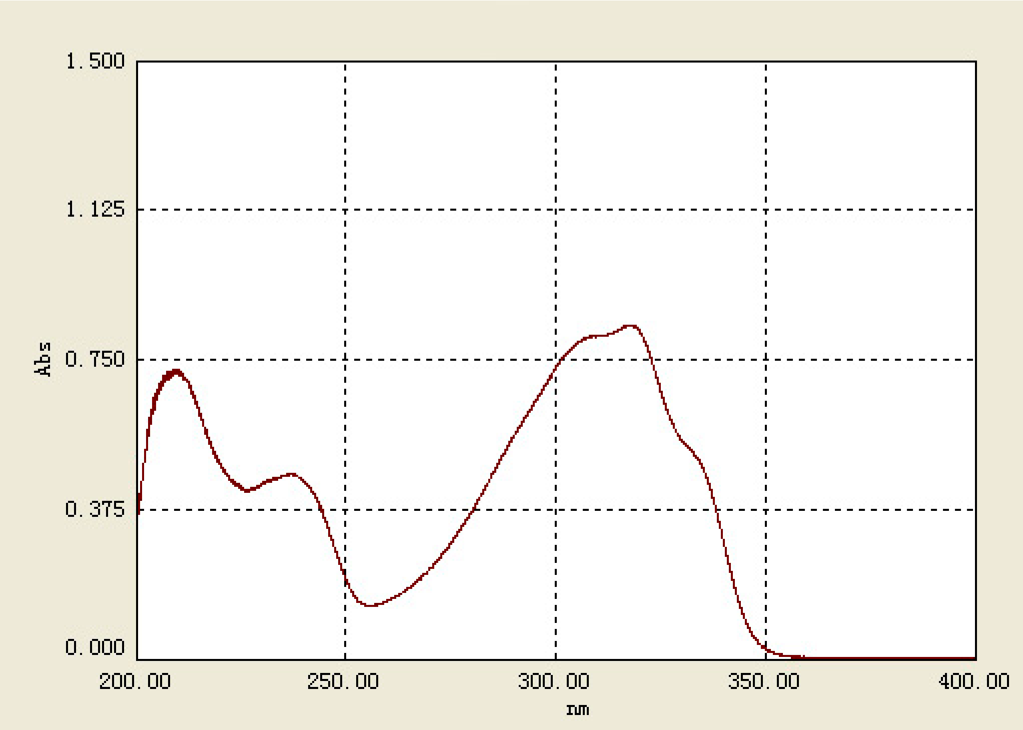

Figure 1. UV spectrum of 1.

Concentration: 0.0100 mg·mL^{-1} (methanol).

**λ (nm)**	**Abs**
209.2	0.727
236.8	0.467
317.4	0.840

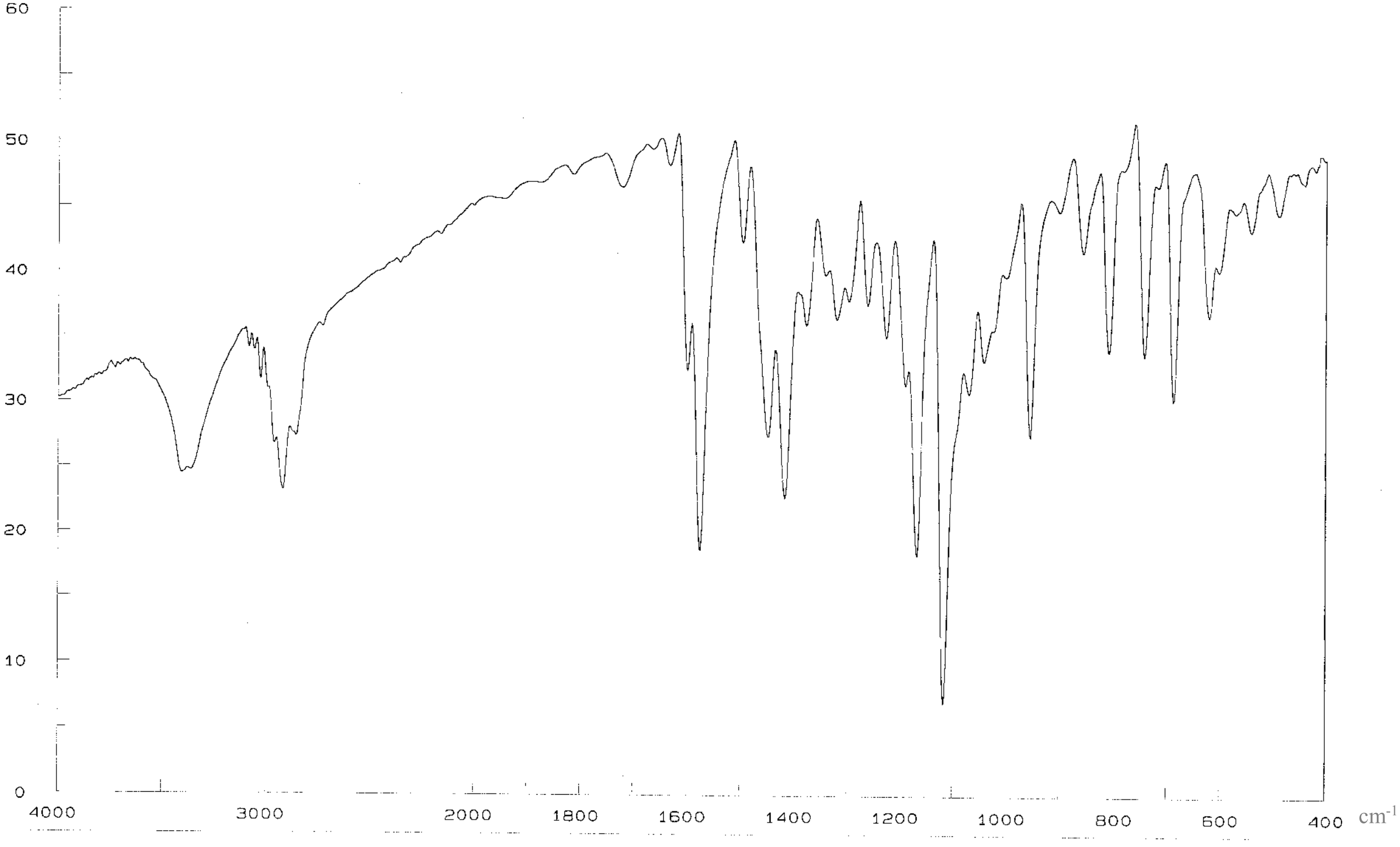

Figure S2. IR spectrum of 1.

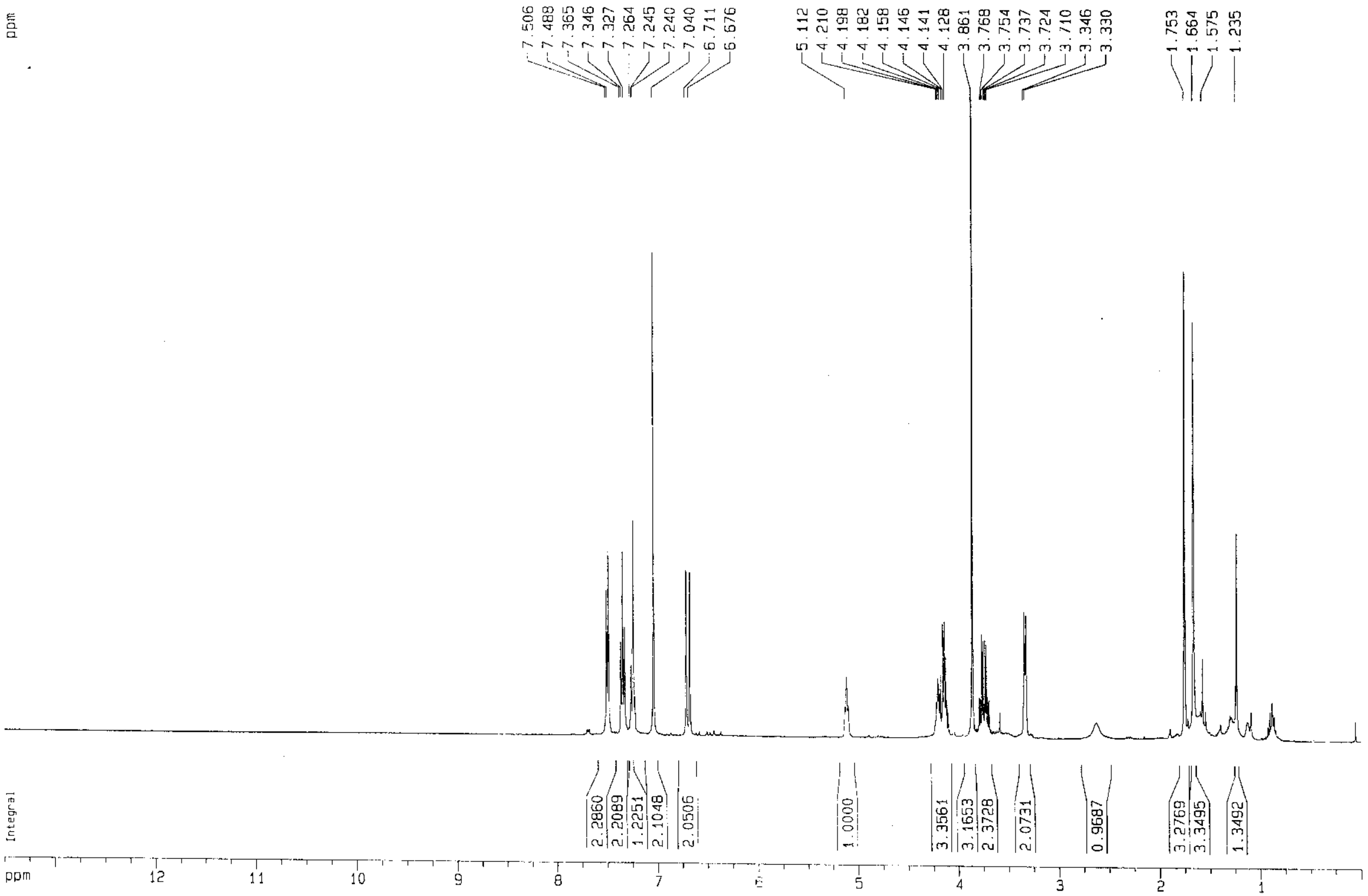

Figure S3. ^{1}H-NMR spectrum of 1 (400 MHz, in $CDCl_3$).

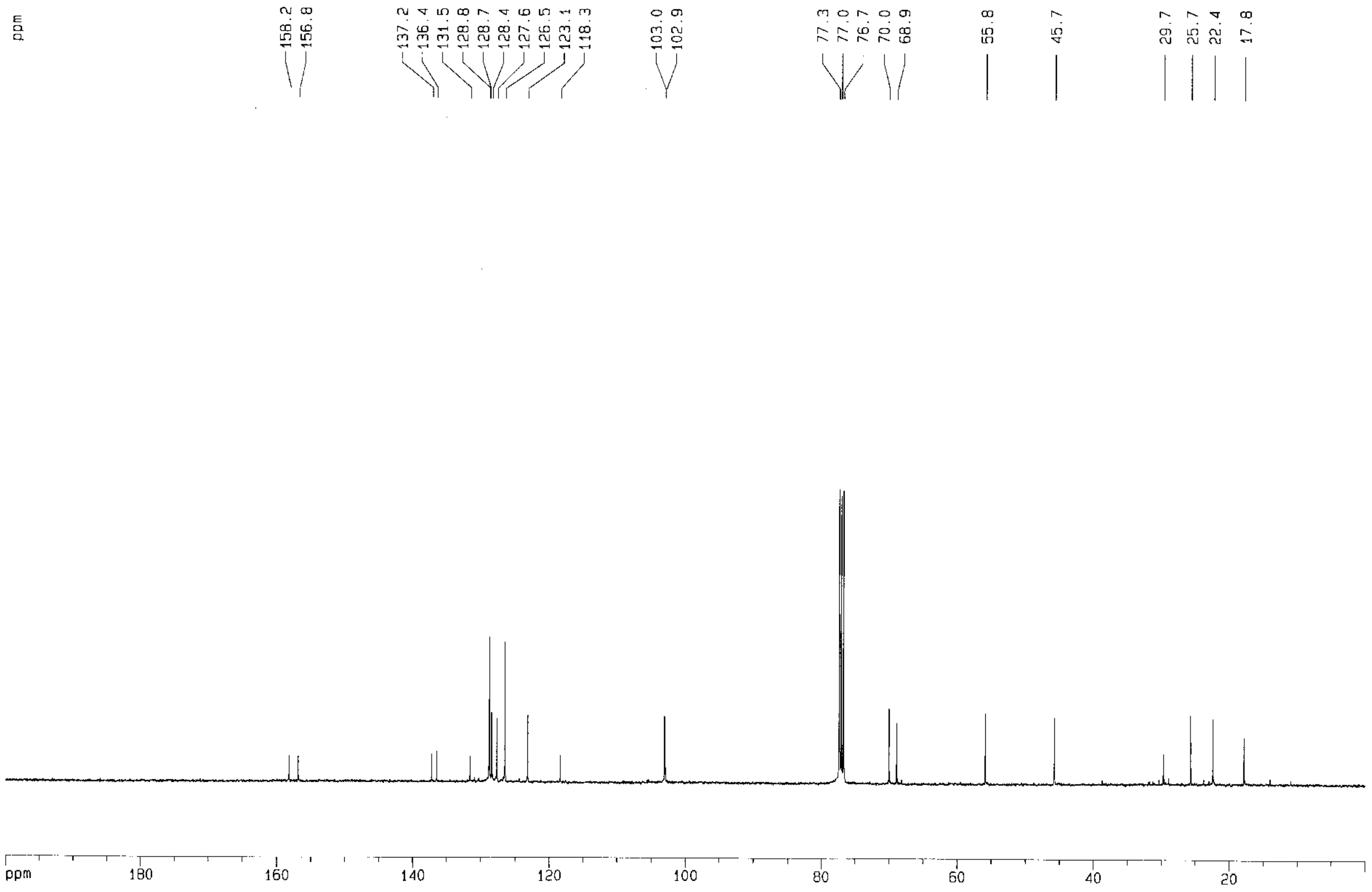

Figure S4. ^{13}C-NMR spectrum of 1 (100 MHz, in CDCl3).

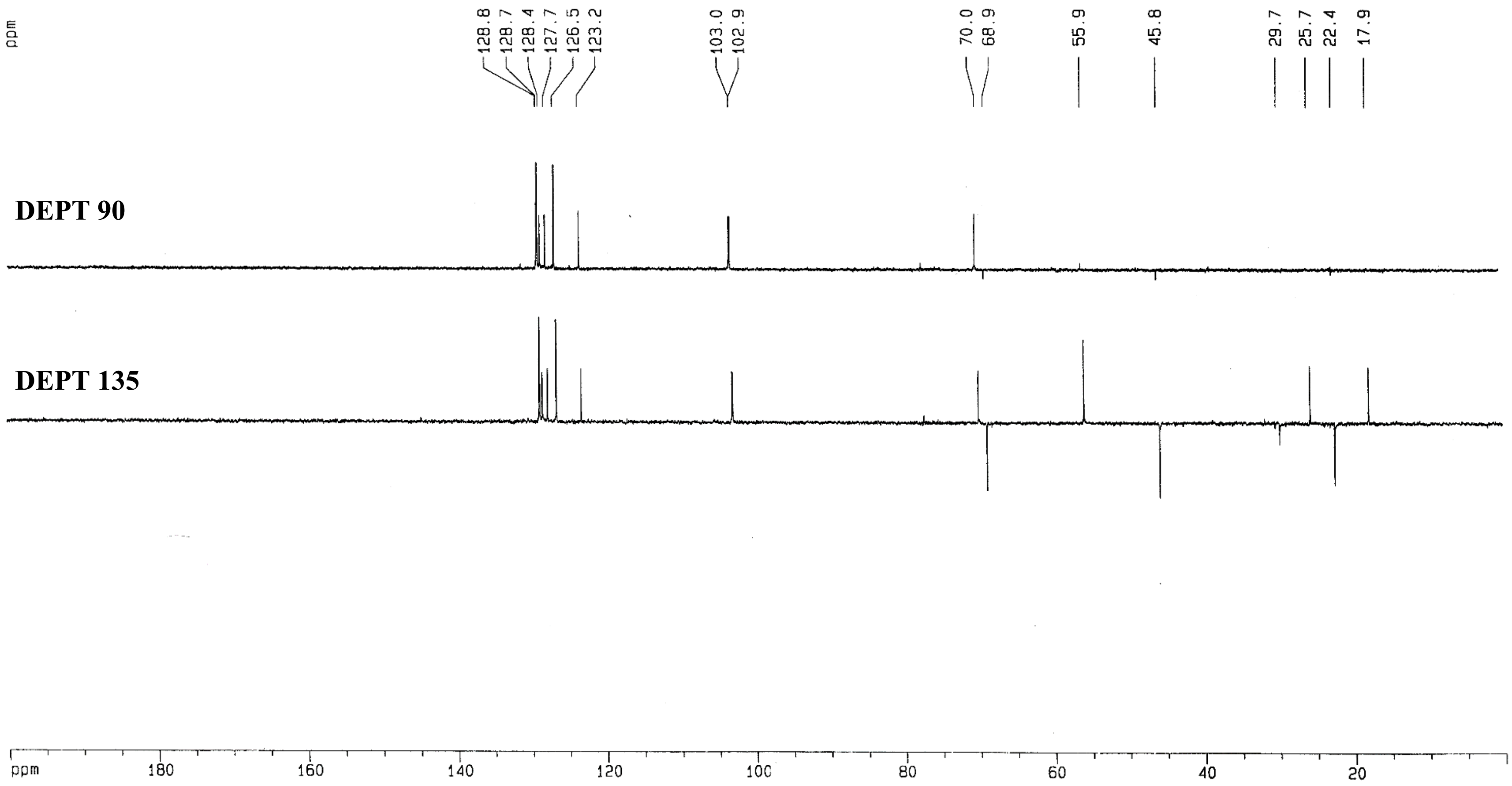

Figure S5. DEPT spectrum of 1 (100 MHz, in $CDCl_3$).

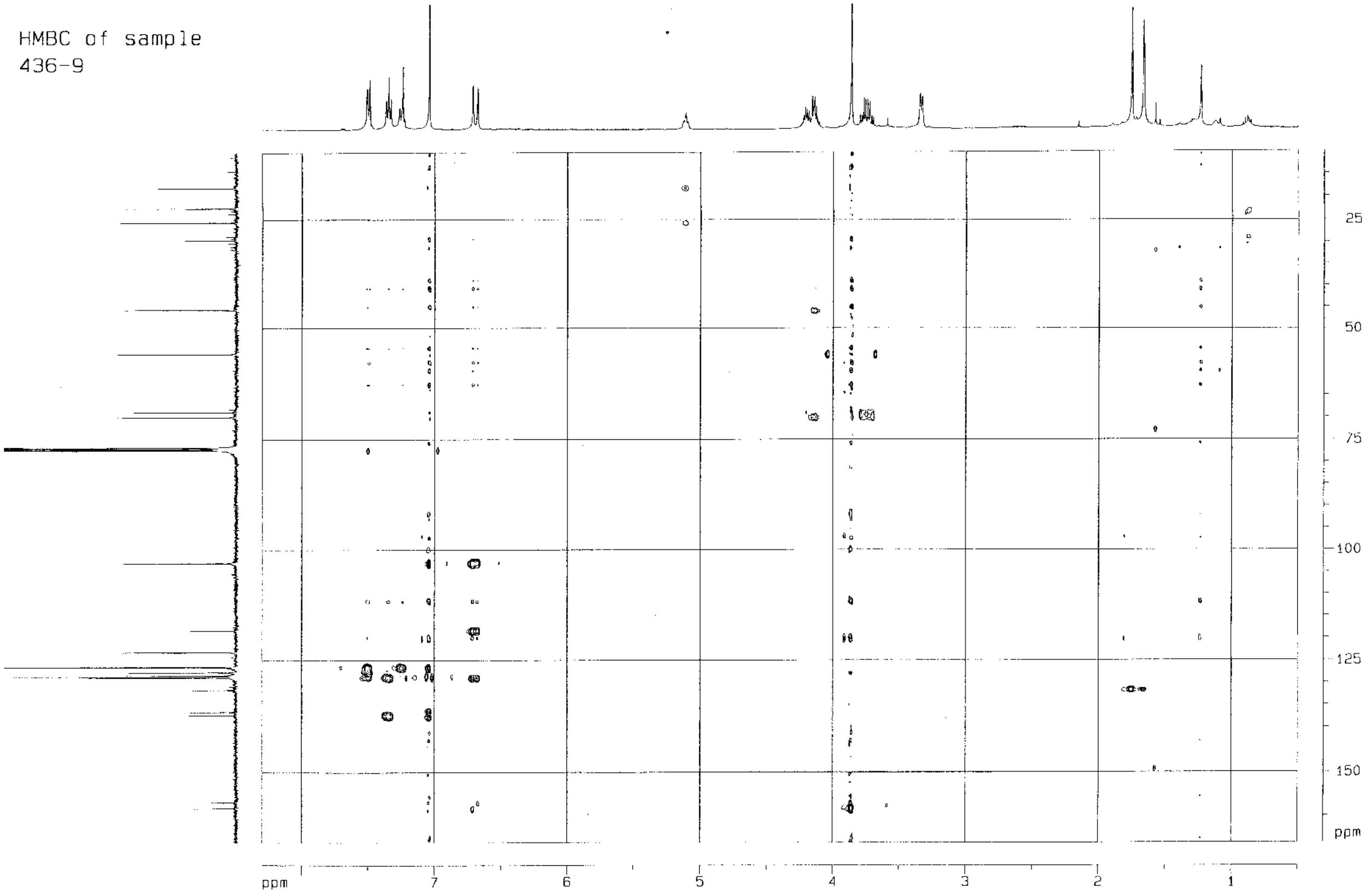

Figure 6. HMBC spectrum of 1 (in $CDCl_3$).

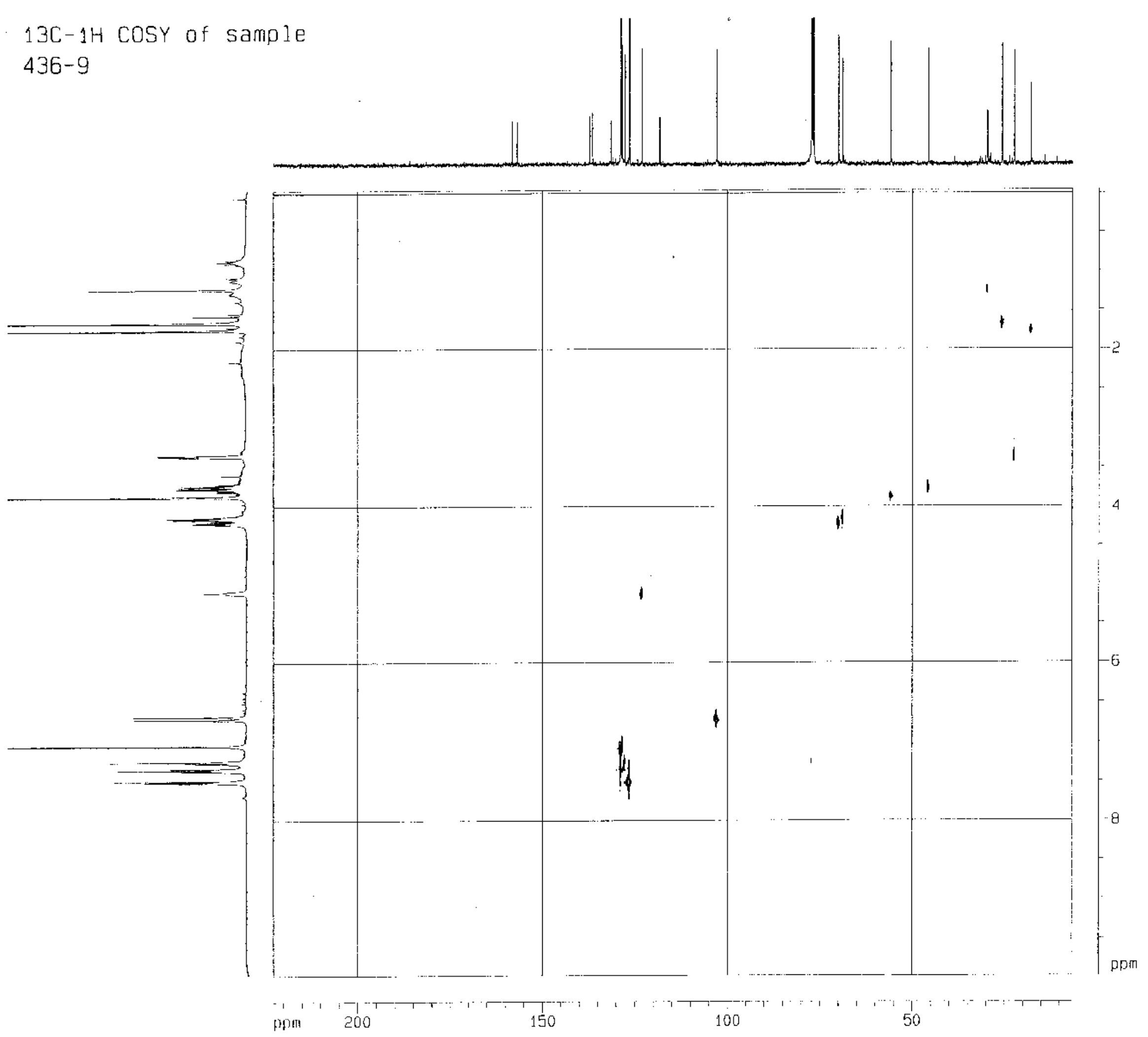

Figure S7. ^{13}C-^{1}H COSY spectrum of 1 (in $CDCl_3$).

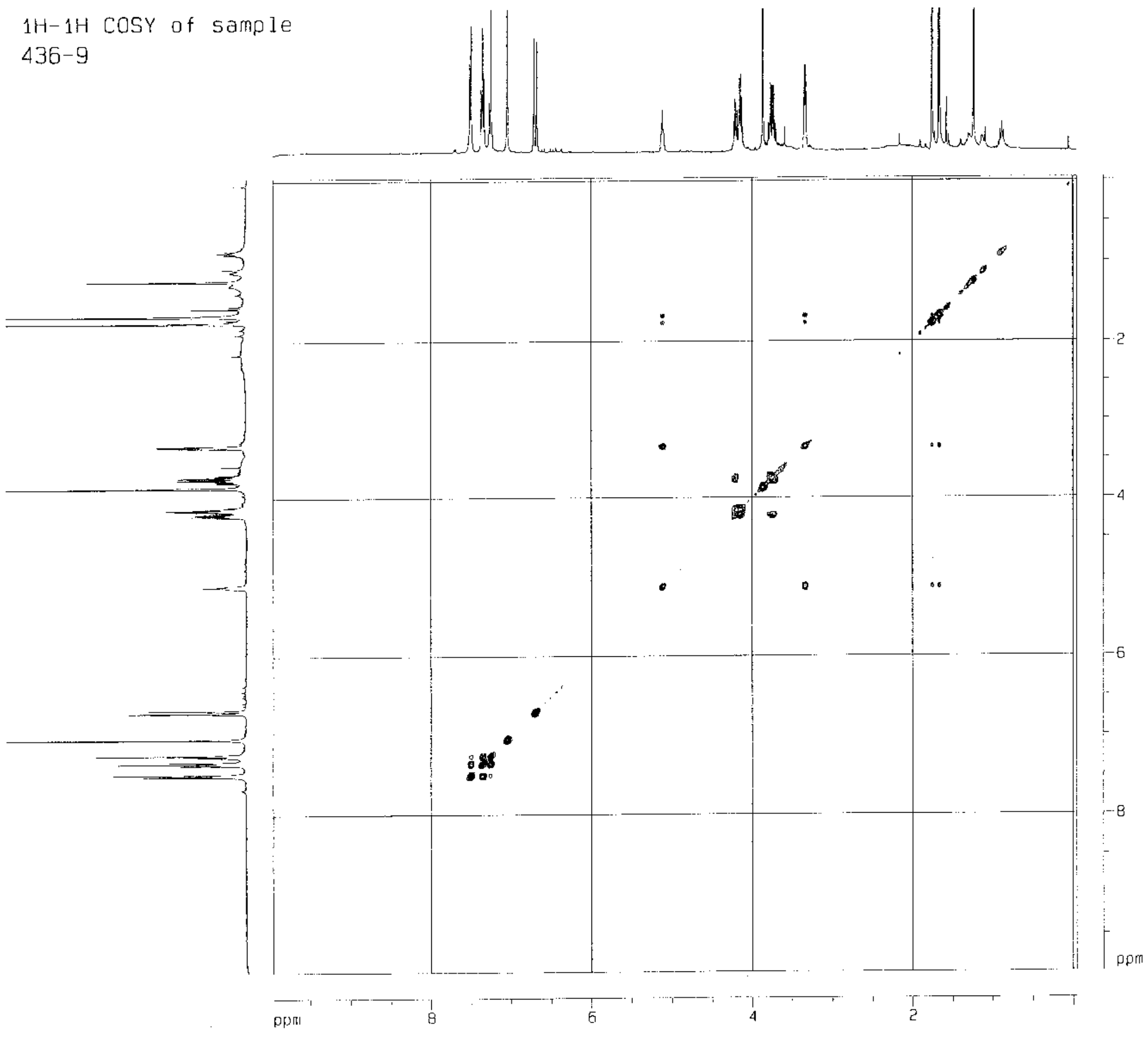

Figure S8. ^{1}H -^{1}H COSY spectrum of 1 (in $CDCl_3$).

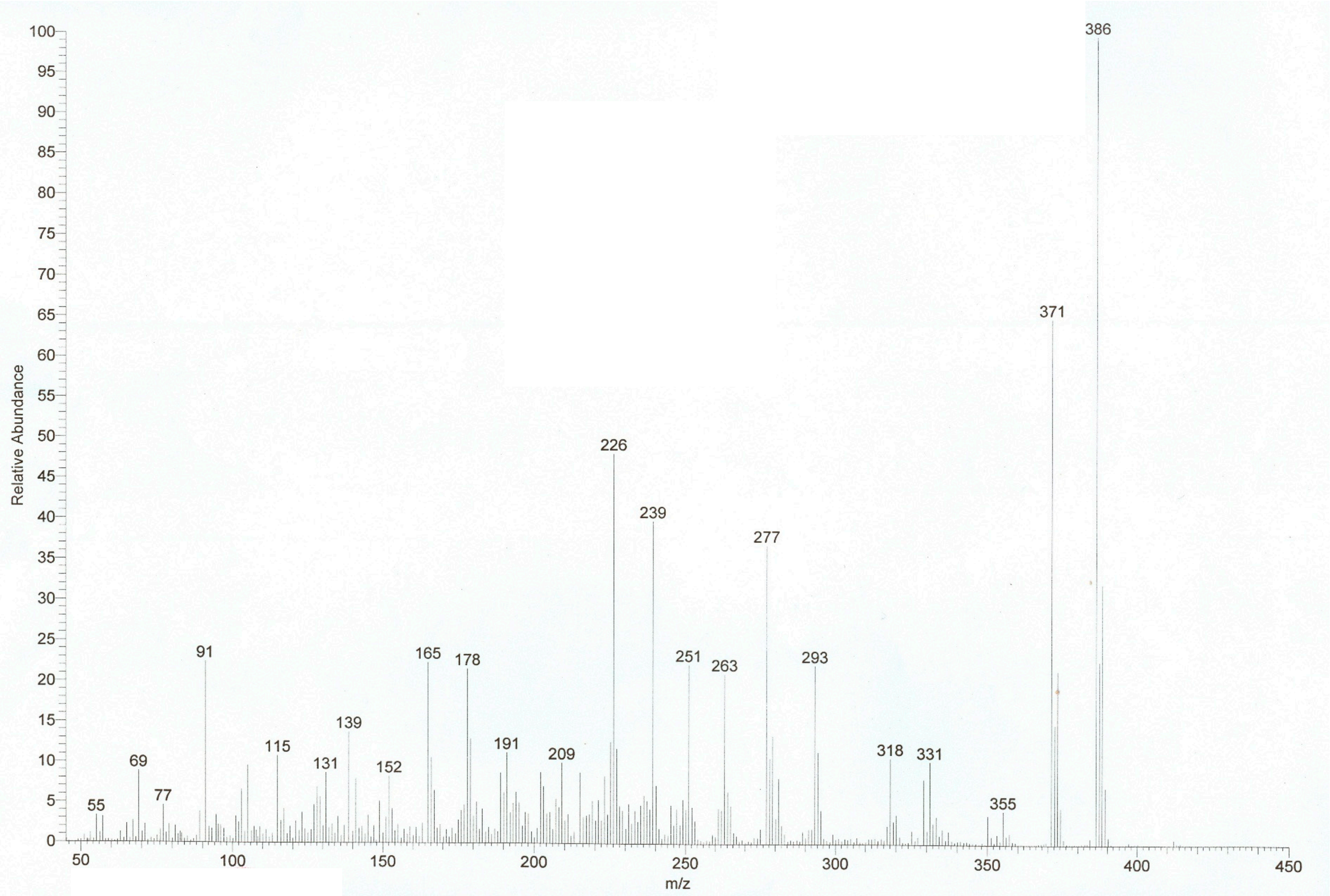

Figure S9. EI MS spectrum of 1.

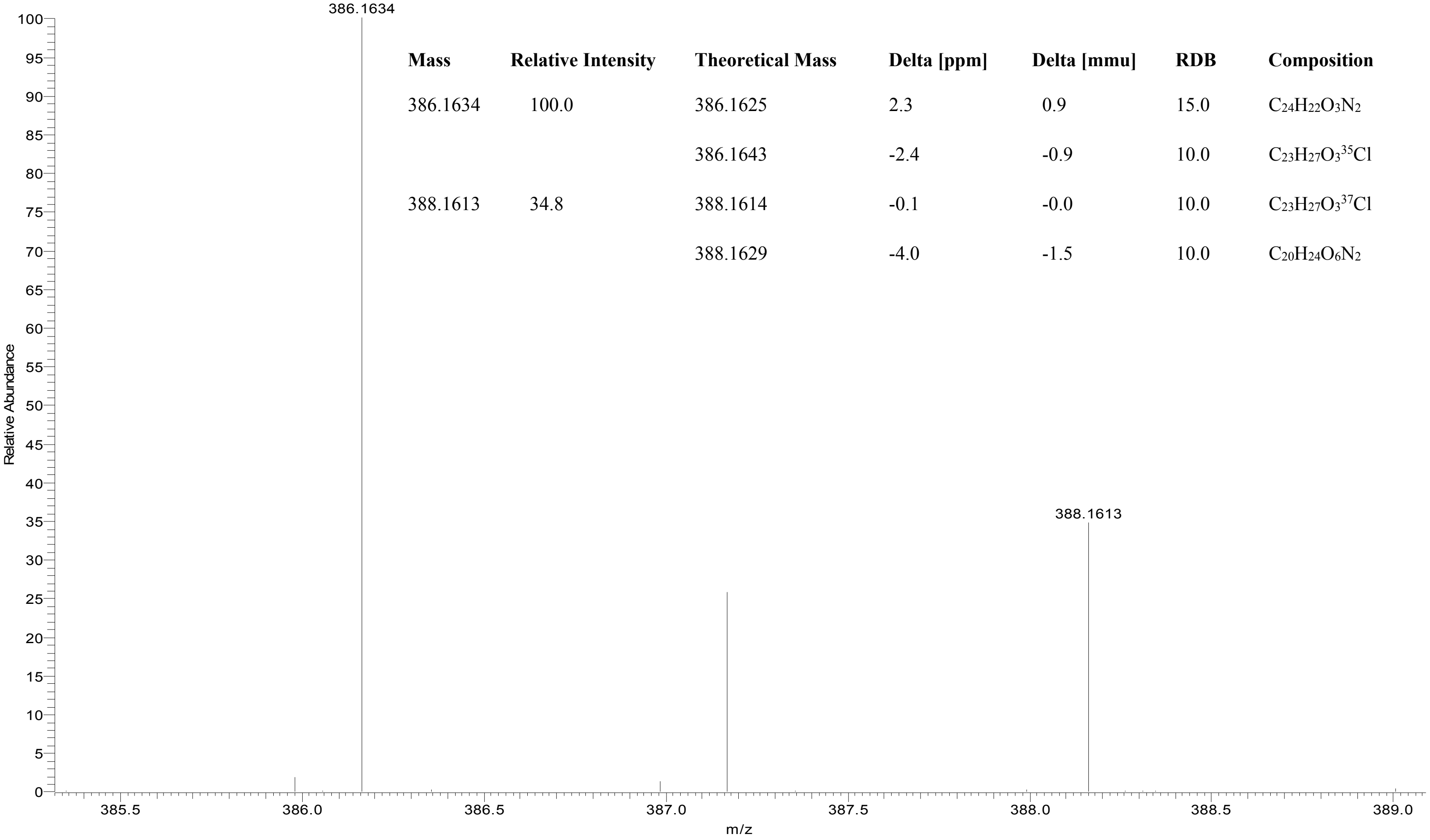

Mass	Relative Intensity	Theoretical Mass	Delta [ppm]	Delta [mmu]	RDB	Composition
386.1634	100.0	386.1625	2.3	0.9	15.0	$C_{24}H_{22}O_3N_2$
		386.1643	-2.4	-0.9	10.0	$C_{23}H_{27}O_3{}^{35}Cl$
388.1613	34.8	388.1614	-0.1	-0.0	10.0	$C_{23}H_{27}O_3{}^{37}Cl$
		388.1629	-4.0	-1.5	10.0	$C_{20}H_{24}O_6N_2$

Figure S10. HR EIMS spectrum of 1.

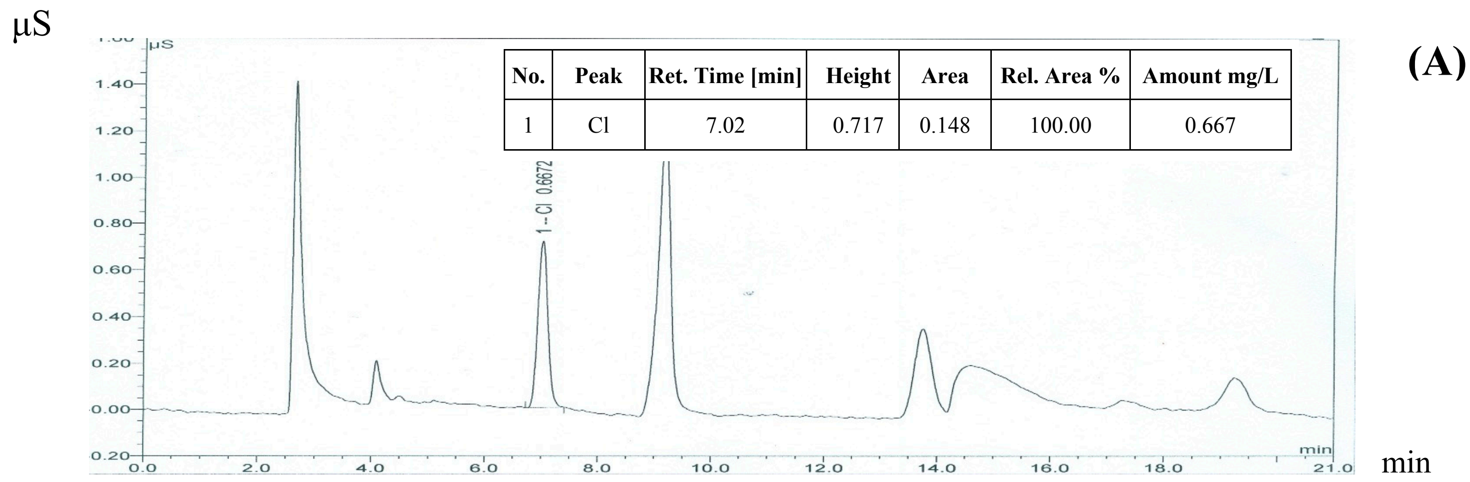

(A)

No.	Peak	Ret. Time [min]	Height	Area	Rel. Area %	Amount mg/L
1	Cl	7.02	0.717	0.148	100.00	0.667

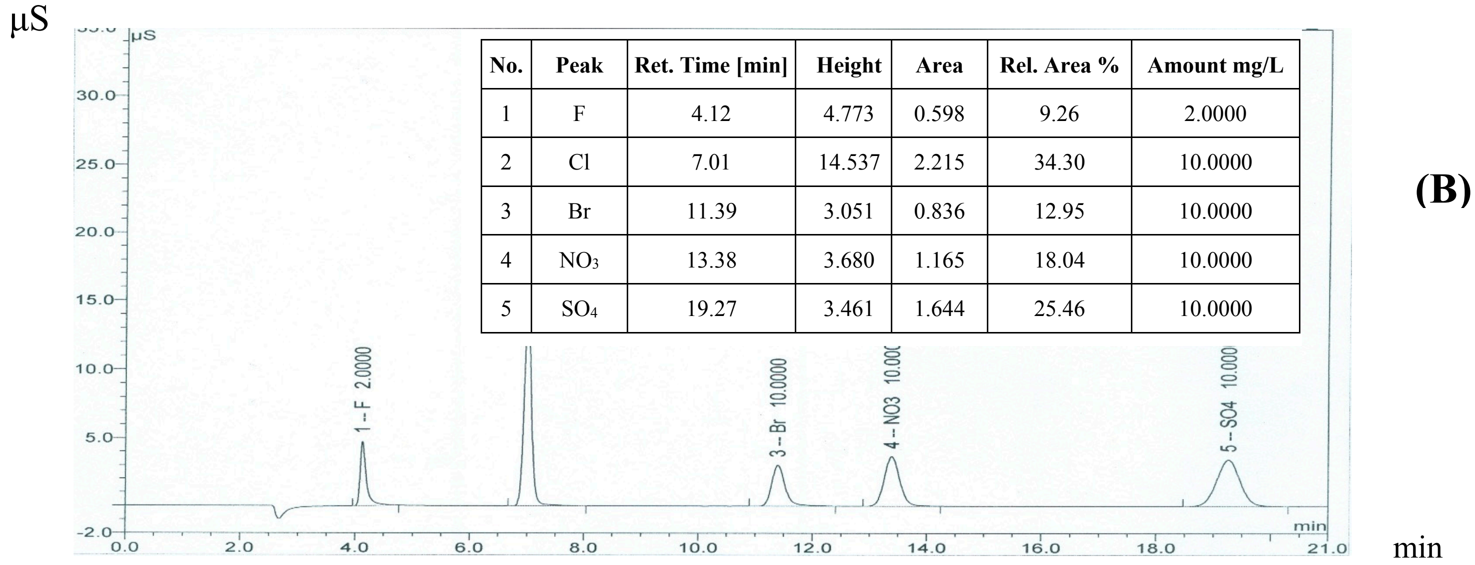

No.	Peak	Ret. Time [min]	Height	Area	Rel. Area %	Amount mg/L
1	F	4.12	4.773	0.598	9.26	2.0000
2	Cl	7.01	14.537	2.215	34.30	10.0000
3	Br	11.39	3.051	0.836	12.95	10.0000
4	NO_3	13.38	3.680	1.165	18.04	10.0000
5	SO_4	19.27	3.461	1.644	25.46	10.0000

(B)

Figure S11. Ion chromatograms for chlorine determination of 1 (EN 14582-2007). (**A**) compound 1; (**B**) mixed standards.
